# Risk of Reactivation of Hepatitis B Virus (HBV) and Tuberculosis (TB) and Complications of Hepatitis C Virus (HCV) Following Tocilizumab Therapy: A Systematic Review to Inform Risk Assessment in the COVID-19 Era

**DOI:** 10.3389/fmed.2021.706482

**Published:** 2021-08-20

**Authors:** Cori Campbell, Monique I. Andersson, M. Azim Ansari, Olivia Moswela, Siraj A. Misbah, Paul Klenerman, Philippa C. Matthews

**Affiliations:** ^1^Nuffield Department of Medicine, University of Oxford, Medawar Building for Pathogen Research, Oxford, United Kingdom; ^2^Department of Infectious Diseases and Microbiology, Oxford University Hospitals NHS Foundation Trust, John Radcliffe Hospital, Oxford, United Kingdom; ^3^Nuffield Department of Clinical Laboratory Sciences, University of Oxford, Oxford, United Kingdom; ^4^Wellcome Centre for Human Genetics, University of Oxford, Oxford, United Kingdom; ^5^Pharmacy Department, Oxford University Hospitals NHS Foundation Trust, John Radcliffe Hospital, Oxford, United Kingdom; ^6^Department of Clinical Immunology, Oxford University Hospitals NHS Foundation Trust, John Radcliffe Hospital, Oxford, United Kingdom

**Keywords:** tocilizumab, biologic, SARS-CoV-2, COVID-19, infection, reactivation, hepatitis B virus, tuberculosis

## Abstract

**Objectives:** Tocilizumab (TCZ), an IL-6 receptor antagonist, is used in the treatment of severe COVID-19 caused by infection with SARS-CoV-2. However, unintended consequences of TCZ therapy include reactivation of tuberculosis (TB) or hepatitis B virus (HBV), and worsening of hepatitis C virus (HCV). We set out to assimilate existing data for these complications, in order to help inform evidence-based risk assessments for the use of TCZ, and thus to reduce the risk of serious but preventable complications.

**Methods:** We searched the global WHO database of Individual Case Safety Reports (ICSRs) and adverse drug reactions (ADRs) (“VigiBase”) and undertook a systematic literature review, in accordance with PRISMA guidelines. We generated mean cumulative incidence estimates for infection complications.

**Results:** Mean cumulative incidence of HBV and TB were 3.3 and 4.3%, respectively, in patients receiving TCZ. Insufficient data were available to generate estimates for HCV. These estimates derive from heterogeneous studies pre-dating SARS-CoV-2, with differing epidemiology and varied approaches to screening and prophylaxis, so formal meta-analysis was not possible.

**Conclusions:** We underline the need for careful individual risk assessment prior to TCZ prescription, and present an algorithm to guide clinical stratification. There is an urgent need for ongoing collation of safety data as TCZ therapy is used in COVID.

## Introduction

**Figure 1:**
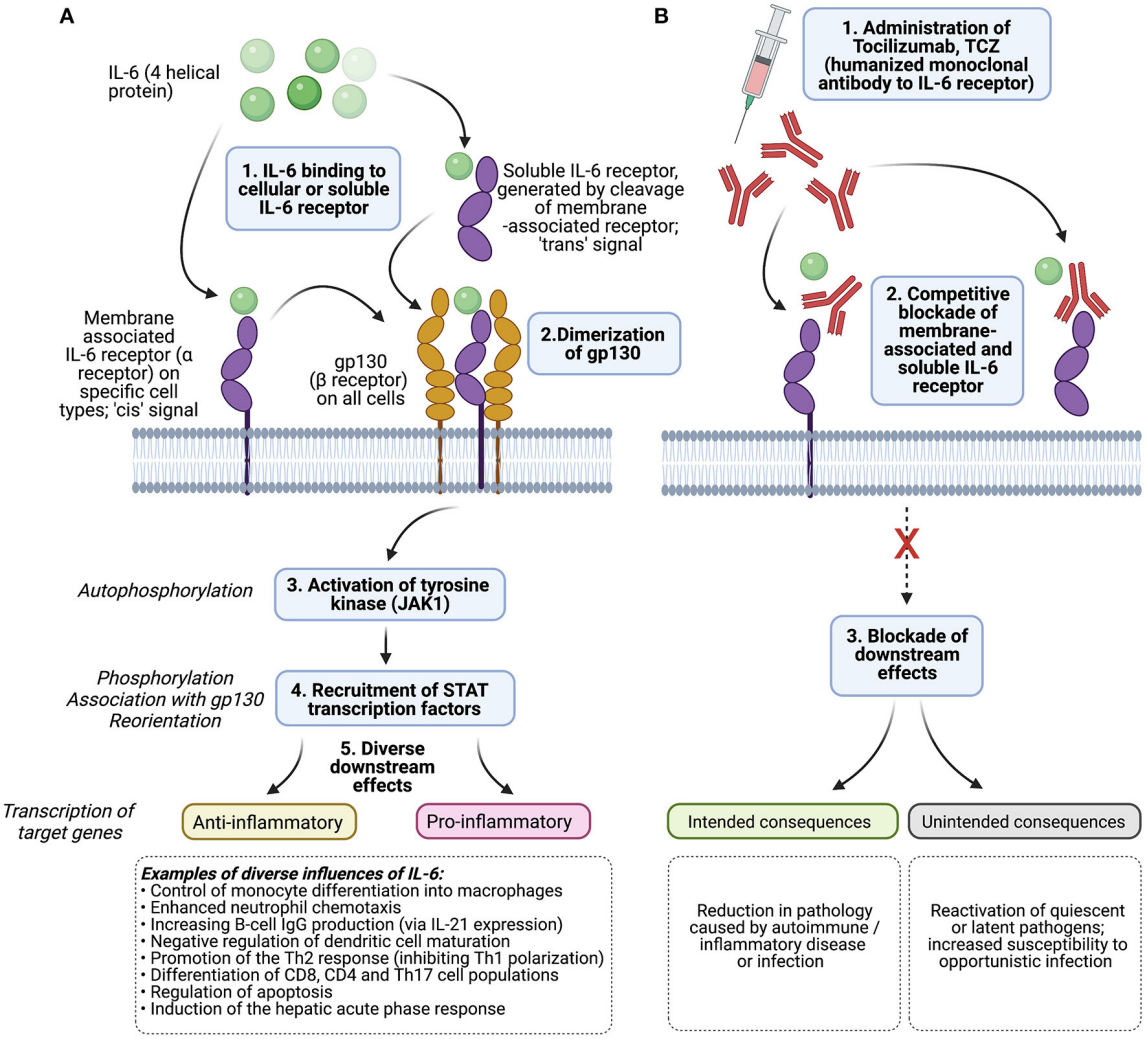
Diagram to show **(A)** the mechanism of action of IL-6 and **(B)** the influence of tocilizumab, TCZ. The influence of IL-6 in viral infection is reviewed by Rose-John et al. (1) and Velazquez-Salinas et al. (2). gp130 is expressed on all cells in the body, IL-6 receptor is expressed by specific cells only (hepatocytes, monocytes/macrophages, CD4+ T cells, some epithelial cells). Cis- (“classical”) signalling is mediated through cell-associated IL-6 receptor, and associated with housekeeping, while trans-signalling describes the pathway associated with free soluble IL-6 receptor which may be more relevant in response to infection. Dimerization of gp130 causes a cascade starting with the autophosphorylation of Janus Kinase 1 (JAK-1). STAT, signal transducer and activator of transcription. Created with BioRender.com.

IL-6 is an immunomodulatory cytokine, performing diverse roles in homeostasis and in the outcomes of infectious, inflammatory and autoimmune disease (Figure 1A) (1). Elevated levels are associated with immune response to infection (2), and also with metabolic and cardiovascular risk (3). Tocilizumab (TCZ) is a “biologic” agent, a recombinant humanised IgG1 monoclonal antibody that competitively blocks both the soluble and membrane-bound forms of the interleukin-6 (IL-6) receptor (Figure 1B). Licenced for the treatment of rheumatoid arthritis, juvenile idiopathic arthritis, giant cell arteritis (4, 5), and for the management of a cytokine storm caused by CAR-T therapy, it is also used off-label for the treatment of other refractory autoimmune or inflammatory disease.

The syndrome of COVID-19, caused by infection with SARS-CoV-2, can be characterised by a “cytokine storm” associated with a severe systemic inflammatory response syndrome, immune dysregulation and a pro-thrombotic state. Elevated IL-6 levels have been associated with the need for invasive mechanical ventilation in these patients (6). For this reason, TCZ has appeal as a therapeutic agent, and multiple studies have set out to determine its role in COVID-19 patients (7). Several clinical studies have reported a benefit [e.g., (8–11)], and the large “Randomised Evaluation of COVID-19 therapy” (RECOVERY) trial reported significantly reduced rates of death and other adverse COVID-19 outcomes in patients receiving TCZ as compared to those receiving usual care (10). Other smaller studies have reported modest improvements in length of hospital stay without reducing deaths (12), while others did not find significantly improved outcomes (13) or even identify possible harm (14). However, a meta-analysis undertaken in January 2021, which included 71 studies (of which 6 were randomised trials), reported overall improved survival associated with TCZ therapy (adjusted mortality risk HR 0.52) (15).

Expert recommendations suggest consideration of a single dose of TCZ (8 mg/kg of actual body weight, up to a maximum of 800 mg) in individuals with respiratory failure secondary to COVID-19 admitted to a critical care environment (16), or with signs of severe or critical disease and elevated markers of systemic inflammation (17). TCZ is recommended in addition to dexamethasone, which has been widely adopted as standard of care based on RECOVERY data (18). TCZ half life is dose-dependent and estimated at 8–14 days (19); some recommendations suggest a repeat dose after 12–24 h if there has not been sufficient clinical improvement (20).

**Figure 2:**
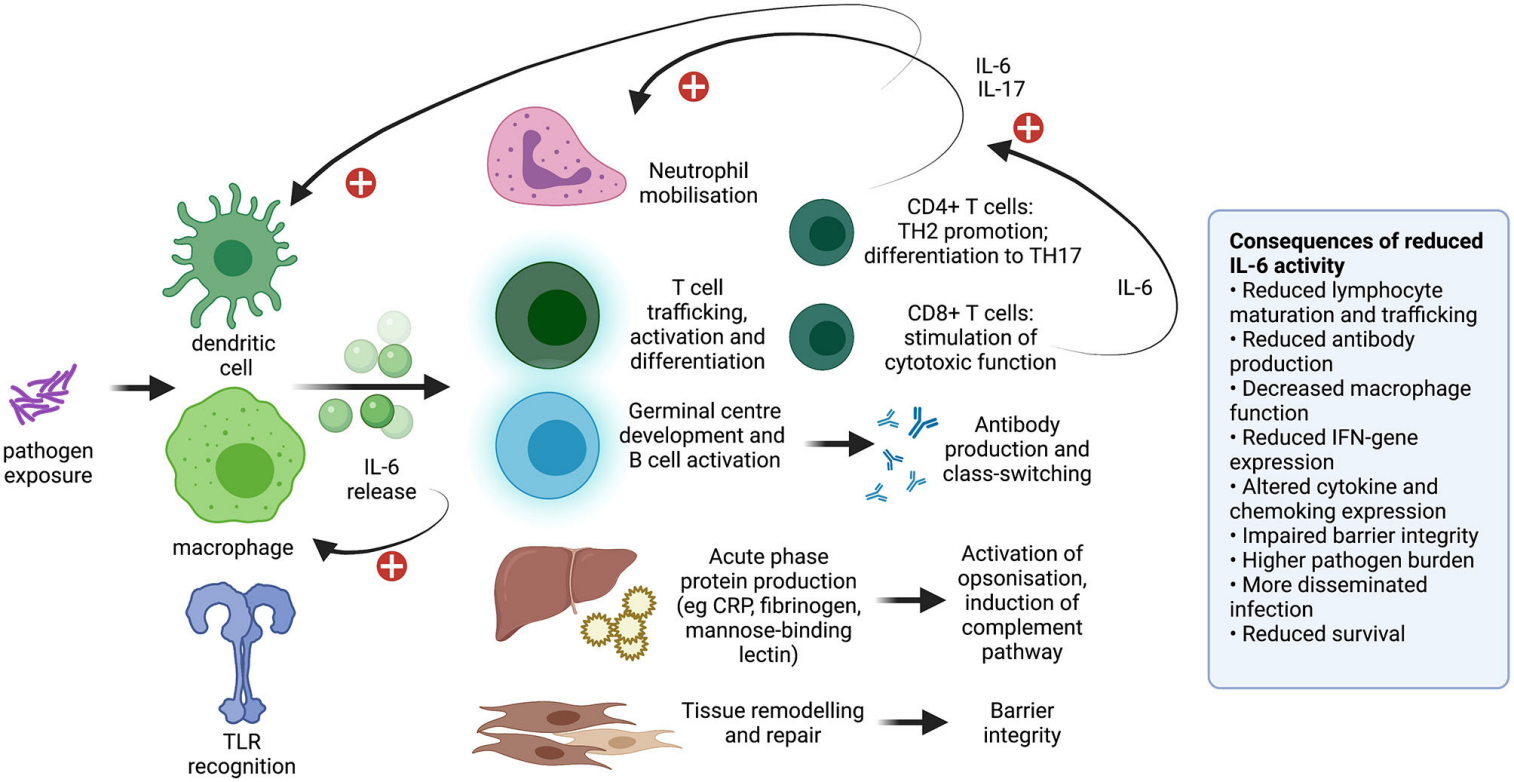
Diagram to show the diverse influence of IL-6 on the immune system. Impact includes monocyte differentiation into macrophages, co-ordination of the neutrophil and lymphocyte response, production of acute phase proteins, and tissue remodelling and repair. Consequences of reduced IL-6 activity have been observed in animal models (27–35) and in IL-6 blockade using monoclonal antibodies including TCZ. This topic is previously reviewed (2). IL, interleukin; TLR, toll like receptor; CRP, C-reactive protein; IFN, interferon. Plus signs indicate a positive feedback loop. Created with BioRender.com.

To date, the safety profile of TCZ in COVID-19 patients is deemed good (7, 13), albeit based on modest numbers, delivery of treatment only in selected clinical settings, and limited periods of follow-up. However, based on experience from the pre-COVID era, TCZ carries a “black box” warning for serious infection events (SIEs; Figure 1B) caused by a range of opportunistic bacterial, mycobacterial, viral and fungal pathogens that may lead to hospitalisation or death. Pooled analyses of the SIE risk associated with the standard dose have estimated incidence at ~5 per 100 patient years (21, 22), similar to other biologic drugs (1), but with higher risk in older adults, in combination with steroids and/or methotrexate (23–25), or with previous biologic therapy (26). Such SIEs can be related to the impairment of IL-6 activity in co-ordinating diverse immune responses, including activation of neutrophils and lymphocytes, acute phase protein production and tissue repair (Figure 2) (2).

TB and Hepatitis B virus (HBV) reactivation are of particular concern for TCZ treatment due to chronicity, latency, and the high global burdens of both infections (Table 1). HCV infection is another blood-borne virus for which outcomes may be complicated by treatment with biologic agents, although this pathogen does not have the potential for latency, and outcomes are thought to be less severe than those of HBV in patients receiving biologic therapies (25).

**Table 1:** Features of Mycobacterium tuberculosis (MTB) and Hepatitis B Virus (HBV) infection pertinent to the use of therapeutic IL-6 blockade.

**Pathogen**	**MTB**	**HBV**
Estimated frequency of carriage in global population	1 in 4 (36)	1 in 3 (37)
Approaches to screening for latent infection/carriage	Interferon-gamma release assay (IGRA) +/– Tuberculin skin test (TST) (38)	HBsAg and HBV DNA negative, but anti-HBc positive^a^ (lowest risk)ORHBsAg positive^b^, with or without detectable HBV DNA (higher risk)
Potential clinical influence of IL-6 blockade	Reactivation, which can result in pulmonary or extra-pulmonary disease (39); potentially life-threatening	Reactivation, which can result in life-threatening liver injury. Complications recognised over a wide time interval, ranging from shortly after starting immunosuppression to after a delay of 1–2 years (40)
Estimated risk of reactivation in individuals prescribed TCZ	Risk classified as “intermediate” (25)	Risk classified as “moderate” or “intermediate” (quantified at 1–10% chance) (40–42)
Recommendations for prophylactic therapy in patients receiving immunosuppressive therapy^c^	Based on clinical risk assessment, consider 3 months of isoniazid (with pyridoxine) and rifampicin OR 6 months of isoniazid (with pyridoxine) (38)	Based on clinical risk assessment, consider prophylactic antiviral therapy with tenofovir (TDF), lamivudine (3TC) or entecavir (ETV) typically recommended for at least 6 months after completion of immunosuppressive therapy (43)

a *The most typical definition of reactivation is in individuals in whom baseline HBsAg (hepatitis B surface antigen) and HBV DNA (viral load) are undetectable but anti-HBc is positive, who then become HBsAg and HBV DNA positive. However, other definitions are also used in which HBsAg is detectable throughout, but reactivation is evidenced by a flare in HBV DNA (viral load rise > 1 log IU/ml) and/or hepatic transaminitis (typically to >2-fold increase above upper limit of normal)*.

b *Individuals testing positive for HBsAg should all be referred to a gastroenterology/hepatology service for risk assessment and to determine eligibility for long-term therapy*.

c *Guidance on prophylactic therapy during periods of immunosuppressive therapy is based on expert assessment, accounting for the nature and proposed duration of treatment*.

Expert opinion and clinical guidelines [including those produced by the National Institute for Health and Care Excellence (NICE)], pre-dating the COVID-19 pandemic, make recommendations about screening and monitoring for TB and HBV infection for patients receiving TCZ, to inform risk stratification, surveillance, and treatment or prophylaxis (24, 25, 38, 44) (Table 1). However, data that can be used to quantify risk are limited, as patients who screened positive for chronic viral hepatitis infection were excluded from pre-marketing authorisation TCZ clinical trials. RECOVERY was primarily undertaken in populations who were carefully screened, and with a low prevalence of exposure to (or active infection with) HBV, TB and HCV, so the absence of reactivation events cannot necessarily be generalised.

Due to the pace of research in a pandemic, large randomised trials evaluating TCZ therapy in COVID patients have not yet been conducted in populations where TB and/or HBV prevalence are high, or with sufficient length of follow up to quantify the risk of reactivation of these infections. Due to small overall numbers of COVID-19 patients treated with TCZ, limited prospective follow-up, and lack of concordance in reported clinical outcomes, guidance recognises the low certainty of the evidence and the pressing need for more data (7, 45).

As more patients are prescribed TCZ for COVID-19, there is an urgent need for data from various populations to inform risk stratification and management of those who are at elevated risk of serious infective complications. We therefore set out to collate data for HBV and TB reactivation, and for complications of active HCV infection, to consider how this evidence may apply to COVID-19 patients, to guide risk appraisal in clinical practise, and to highlight where further action is needed.

## Methods

### Literature Search

Our population of interest included individuals at potential risk of TB, HBV, or HCV complications, who were receiving TCZ based on any clinical indication.

We searched the global WHO database of Individual Case Safety Reports (ICSRs) / adverse drug reactions (ADRs) (“VigiBase”) on 21st February 2021 (46); search terms are presented in Supplementary Table 1. We set out to identify studies reporting the risk of TB, HBV, or HCV complications in individuals taking TCZ as compared to those not receiving TCZ. We undertook a systematic literature review (on 25 February 2021), searching Medline, Embase, and Web of Science databases using search terms presented in Supplementary Table 2, in accordance with PRISMA guidelines (Supplementary Table 3). We searched all databases from 1 January 2002 to 25 February 2021. We did not apply any further restrictions (specifically, we did not impose any restrictions based on study design, as we intended to produce a comprehensive summary of the evidence surrounding this research question). We included any article (including abstracts) reporting/investigating the risk of adverse HBV, TB and or hepatitis C virus (HCV) outcome(s) associated with TCZ use. Results from each database were combined and deduplicated prior to eligibility screening. Reference lists of relevant systematic reviews/meta-analyses were also searched to identify studies for inclusion.

### Data Extraction and Statistical Analysis

**Figure 3:**
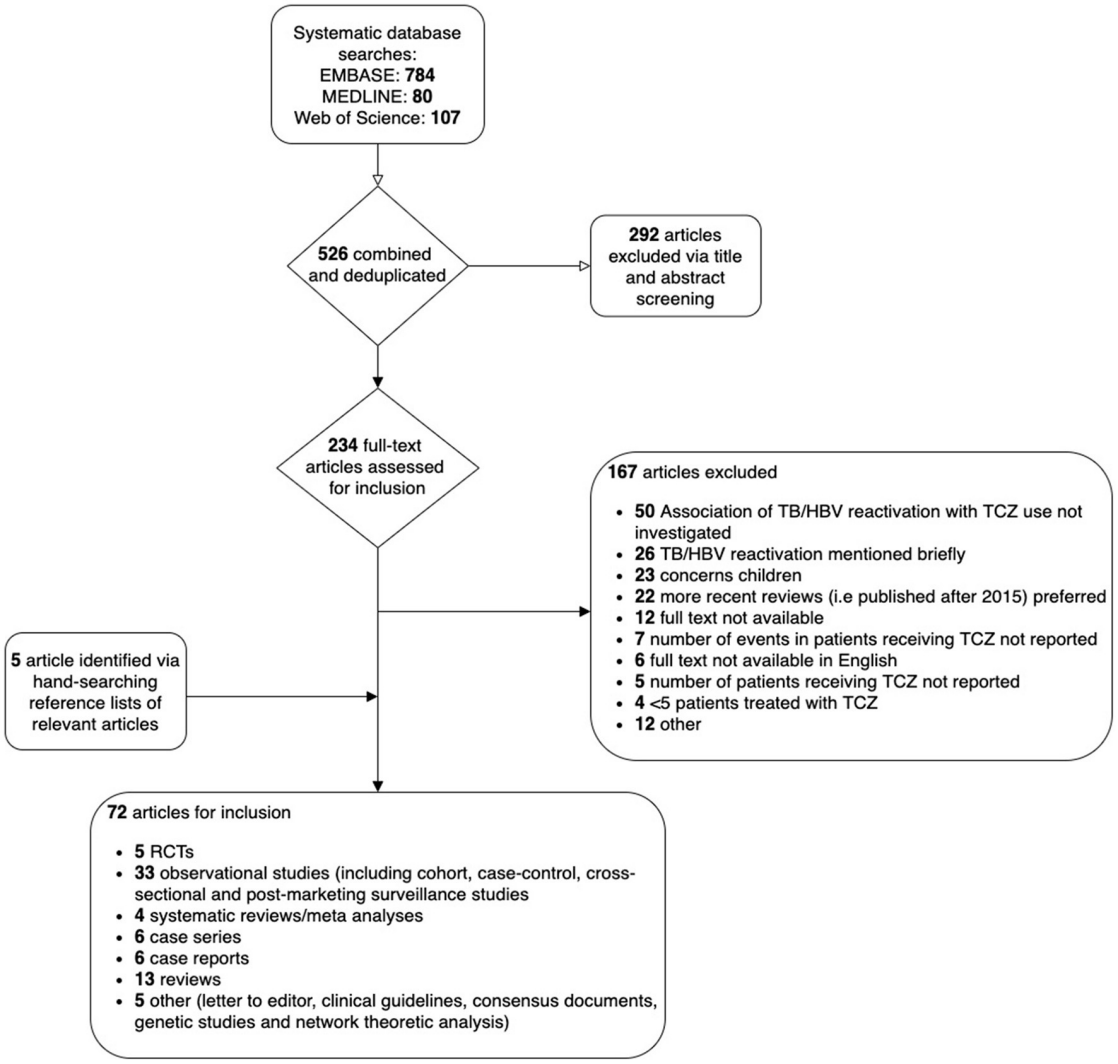
Flow chart to show selection of studies reporting the risk of HB and/or TB reactivation in patients treated with tocilizumab (TCZ) from a systematic literature review.

Studies were screened and were considered eligible for inclusion if they met pre-stipulated criteria, namely that they were observational cohort or post-marketing surveillance studies reporting both (i) the number of patients in the study sample receiving TCZ and (ii) the number of TCZ-treated patients experiencing an outcome of interest (TB or HBV reactivation, or complications of HCV infection) (Figure 3). In the case of ambiguity, a second author reviewed the study and articles were discussed thoroughly until agreement was reached. For each study, we recorded country, publication year, study design, study population, follow-up period, number of participants receiving TCZ, number of TB/HBV reactivation cases, age at baseline (mean/median), and sex. We also collated information pertaining to screening of participants for TB/HBV at baseline, as this is essential to provide context about the population treated, and is a potentially important source of bias.

Our original analysis plan was to undertake meta-analysis to calculate inverse-variance weighted pooled risk estimates for complications pertaining to TB, HBV and HCV, using individual risk estimates from identified studies. However, having collated our dataset, we did not perform meta-analysis, as studies were inconsistent in reporting the number of patients with chronic HBV and/or latent TB infection. This is important as the included studies were undertaken across a diverse range of geographical regions, and therefore HCV, HBV and TB prevalences in the underlying populations from which study samples were drawn varied substantially, with an impact on the estimated risk of reactivation. For example, a large study from a population with low prevalence of HBV and TB infections might report with high confidence that TCZ is not associated with reactivation, as it is unlikely that anyone with those infections was included in the study. Additionally, some studies offered prophylactic treatment to patients who screened positive for TB or HBV before administering TCZ, whilst others did not. Definitions of HBV/TB reactivation endpoints were also inconsistent, with varying lengths of follow-up. Instead, we calculated mean cumulative incidence estimates for HBV and TB reactivations from observational cohort studies. Individual and mean cumulative incidence estimates were displayed in forest plots constructed in R (version 4.0.2) using the “metaphor” package (version 2.4-0 [https://cran.r-project.org/web/packages/metafor/metafor.pdf]). Upper 95% confidence intervals were calculated for studies reporting zero events of interest using the rule of three, whereby the upper limit is approximated using 3/(n+1), as previously described (47). For HCV, data were insufficient for this purpose, and we have summarised data in a narrative form.

## Results

### “Vigibase” WHO Database Reports HBV and TB Reactivation in Patients Prescribed TCZ

The VigiBase database (46) holds 47,205 records for complications of TCZ, of which 14,147 (30%) pertain to “infections and infestations,” with tuberculosis mentioned in 135 cases (0.9% of infections), and HBV in 42 (0.3% of infections) (Supplementary Table 1). However, further interpretation and risk quantification is not possible based on this source, as the database does not contain any denominator data (total number of individuals treated with TCZ therapy), no details of clinical or demographic history, nor the indication/dose/duration of TCZ therapy.

### Systematic Review to Determine the Risk of TB Reactivation in Patients Receiving TCZ Therapy

**Figure 4:**
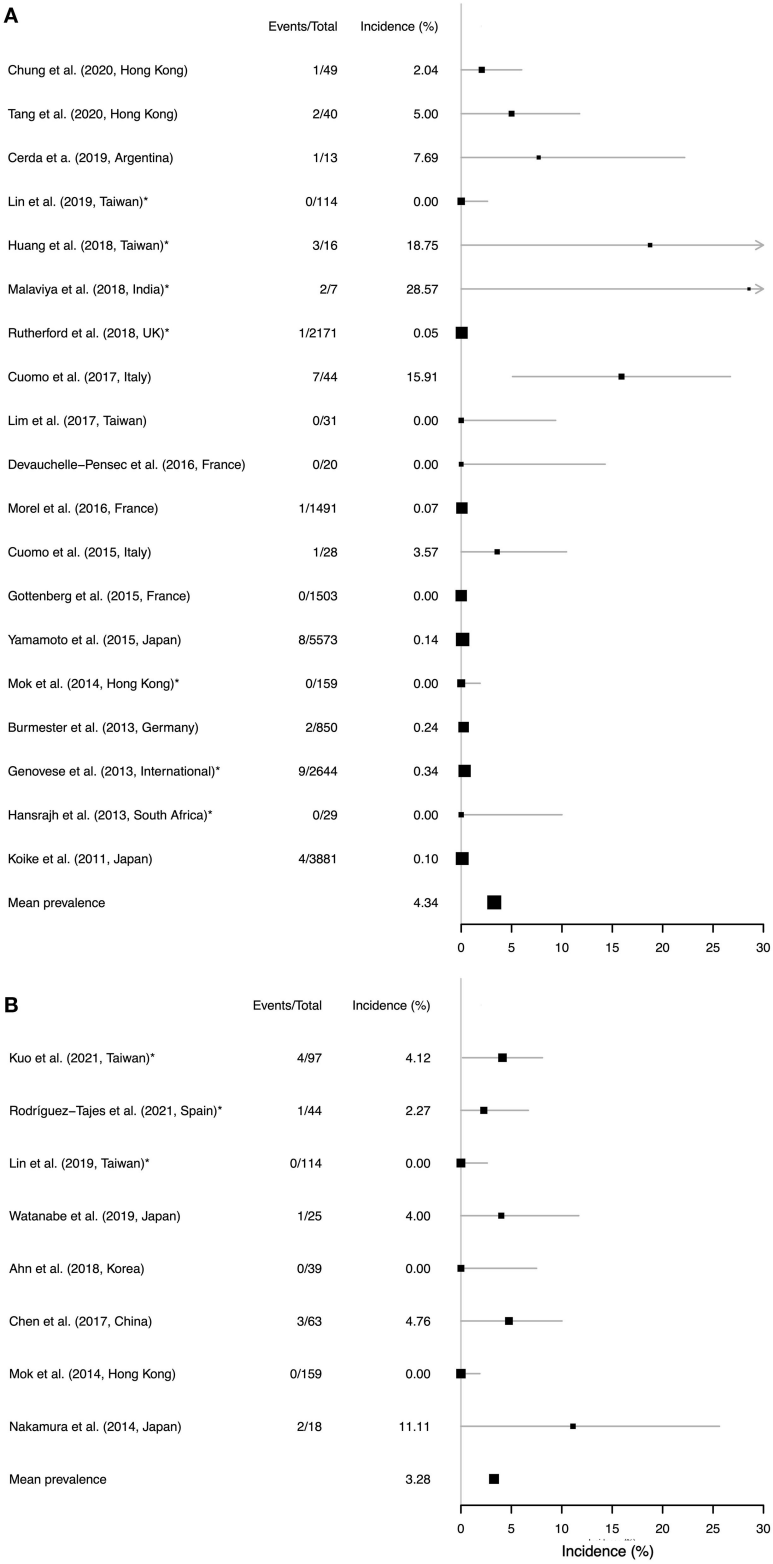
Forest plots to show data from a systematic literature review to identify studies reporting a risk of reactivation of **(A)** TB and **(B)** HBV in individuals treated with TCZ therapy. Studies are ranked in order of follow-up duration. Location of the study is presented for each study, as the baseline prevalence of TB and HBV infection varies substantially by geography. Reactivation incidence (x-axis) shows proportion of events among all those treated over the follow-up period. The size of each point estimate is proportional to cohort size. Asterisks (*) denote studies in which prophylaxis was administered (in **(A)**, TB prophylaxis administered to patients with latent infection; in **(B)**, HBV prophylaxis administered to a proportion of individuals with positive HBV serology). A pooled mean estimate is presented in each panel, but should be interpreted with caution due to the difficulties in combining heterogeneous data from diverse settings. Confidence intervals were estimated using binomial proportion. For studies reporting zero events the upper 95% confidence intervals were calculated using the rule of three, whereby the upper limit is approximated using 3/(n+1).

We identified 19 observational cohort studies in which the incidence of TB reactivation was reported, with a follow up duration of between 24 weeks and 11 years (Figure 4A; Supplementary Table 4). Studies were typically small, with a median size of 49 participants, although six studies reported on data for >1,000 individuals. The incidence of TB reactivation varied from no reported cases (in six studies; Supplementary Table 4), up to 29% (but with wide confidence intervals, 95% CI 5.4, 66.1) in a small Indian study (48). This variation is likely to reflect significant differences in risk between settings according to the population prevalence of latent TB, as well as varied approaches to screening and prophylaxis. Although the Indian study reports risk assessment and prophylaxis, they nevertheless identified reactivation events (48). In contrast, a study in South Africa—another high prevalence setting—recorded zero cases of TB reactivation (49), which is likely to reflect successful chemoprophylaxis. Several studies did not report their approach to screening and prophylaxis, nor did they provide specific information regarding TCZ dosage (Supplementary Table 4), thus making direct comparisons between studies difficult.

A mean cumulative incidence estimate from the available data puts the estimated risk of TB reactivation at 4.3% at a population level, but this figure should be interpreted with caution on the basis of the heterogeneity described above.

### Systematic Review to Determine the Risk of HBV Reactivation in Patients Receiving TCZ Therapy

We identified 8 observational cohort studies in which the risk of HBV reactivation was reported, with a median of 46 study participants (range 18–159); (Figure 4B; Supplementary Table 5). Follow-up was undertaken over periods ranging from 1 month up to 9 years, with no cases of HBV reactivation reported in three cohorts, but up to as high as 13% in a Japanese cohort (50). Baseline prevalence of HBsAg and anti-HBc varied between cohorts, and approaches to screening and prophylaxis were undertaken differently between settings, with a more rigorous approach in some centres that may explain the lower incidence of reactivation events. A mean cumulative incidence estimate from the available data puts the estimated risk of HBV reactivation at 3.3% at a population level, but as for TB these figures should be interpreted with caution and individual risk assessment is key.

### Systematic Review to Determine the Risk of Complications of HCV Infection in Patients Receiving TCZ Therapy

We identified six studies which investigated or discussed the risk of HCV complications associated with TCZ use (Supplementary Table 6). Most studies reported low risks of HCV flare associated with TCZ use, but nevertheless called for future investigations and screening of rheumatoid arthritis patients before administration of anti-rheumatic treatment. The scarcity of evidence in this field has been previously highlighted (51).

### Review Articles

**Figure 5:**
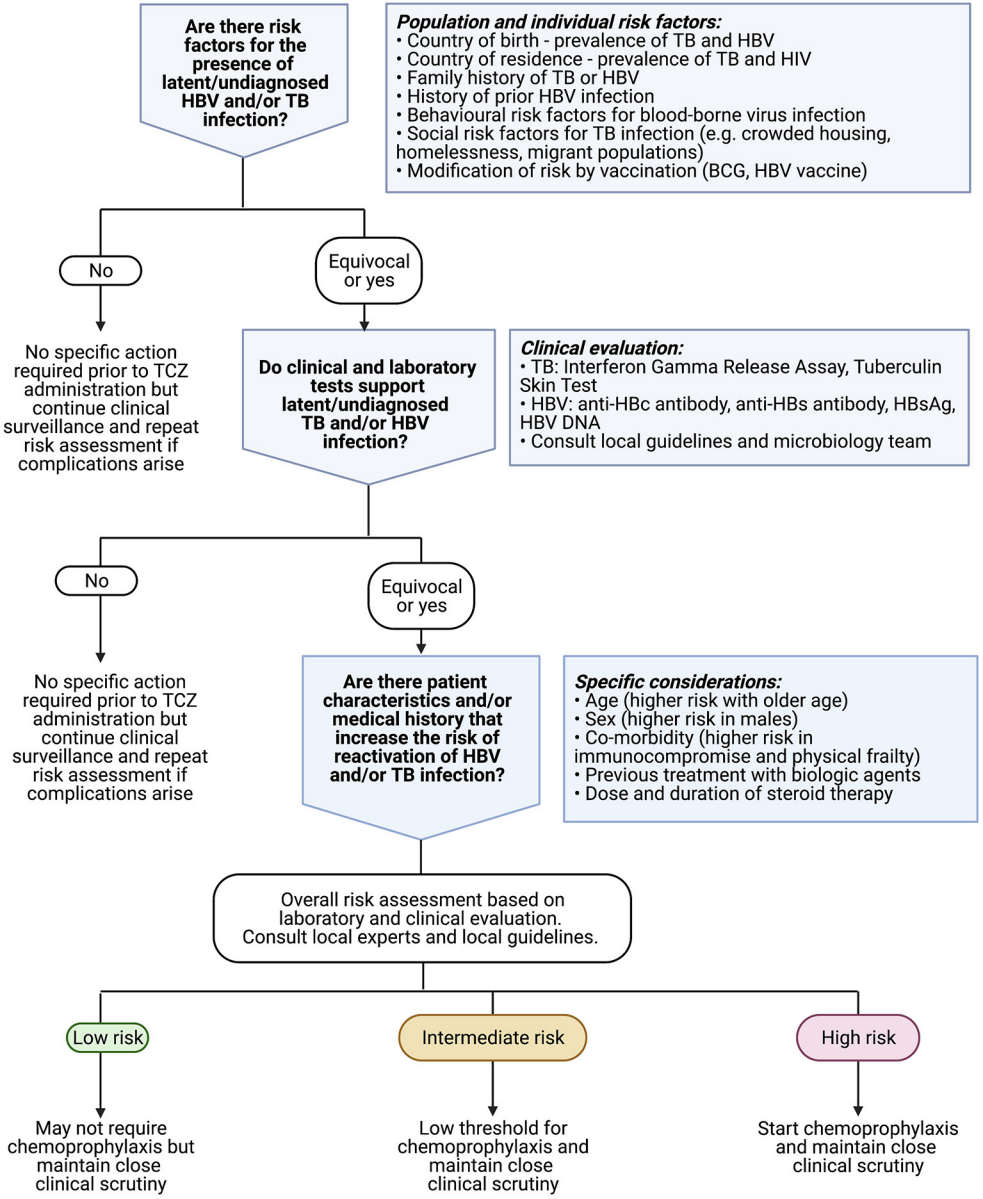
Algorithm to underpin clinical approach to risk assessment for latent or undiagnosed TB or HBV prior to commencing treatment with TCZ. Existing recommendations for anti-cytokine therapy suggest considering antiviral prophylaxis especially in patients who are older in age, male sex, frail, and/or have underlying haematological malignancy (41). Clinicians should also consult relevant local guidelines for use of biologic therapy [e.g., (52)], and consider local epidemiology including vaccination coverage. Created with BioRender.com.

Review and guidance articles are unified in highlighting potential risks of HBV, HCV and TB infection, although with varying risk appraisal (Supplementary Table 7). Most regard the risk of HBV or TB reactivation associated with TCZ therapy as “moderate” or “intermediate.” Typically, screening is recommended for HBV and TB (±HCV) prior to starting any biologic therapy, and prophylaxis is recommended in those deemed to be at the highest risk of complications (summarised in Figure 5).

### Meta-Analysis

We considered undertaking meta-analysis to generate a pooled estimate of reactivation rates with potential comparison to rates “control” groups (those not receiving TCZ therapy), but unfortunately inclusion criteria and study population characteristics (including control group demographics) varied across cohorts and were not consistently reported. Therefore, it would not be robust or appropriate to calculate ratios or comparative statistics that compare infective complications across studies. Furthermore, sample sizes were small, so such an analysis would be underpowered.

### Risk of Bias

Characteristics of the individual studies may have contributed to bias. Populations studied may be skewed against reactivation events (if studies are not conducted in high risk populations, or if individual patients at high risk are given prophylaxis or actively excluded from trial participation). Conversely other studies set out to enrich for individuals with prior HBV exposure such that risks for a general population may be over-estimated. Examples of each of these sources of bias are presented with details in Supplementary Table 4 (for TB) and Supplementary Table 5 (for HBV). Durations of follow-up were short in most studies, likely making them insufficient to detect all possible TB or HBV reactivation events. Baseline approach to screening and/or administration of prophylaxis for TB and/or HBV was not always reported, and was inconsistent across studies.

## Discussion

### Summary

TCZ is gaining traction as therapy for patients at the severe end of the clinical COVID-19 spectrum, and has been incorporated into clinical recommendations (16, 17), which will result in its more widespread use in a critically ill patient group at risk of infective complications because of the global prevalence of latent TB and HBV carriage (Table 1). Our summary data demonstrate a small risk of HBV and TB reactivation overall, with estimates of 3 and 4%, respectively, for a population treated with TCZ. However, our ability to quantify these risks and undertake meta-analysis is constrained by limited and heterogeneous data and a lack of appropriate control (TCZ-untreated) comparator groups. These estimates should be considered a preliminary benchmark to inform awareness rather than absolute risk estimates. We therefore highlight the need for more data and the imperative for individualised clinical risk assessment, based on the individual patient and the population setting, to underpin prescription of TCZ therapy (Figure 5).

### Specific Clinical Recommendations

Consensus from existing guidelines recommend screening for carriage of HBV and TB prior to TCZ therapy. However, even where recommendations are established, screening and prophylaxis are often not consistently undertaken (41, 44), and more data are needed to better inform risk assessment and prescription of prophylactic therapy. Chemoprophylactic agents are widely available, can be administered enterally, are cheap, safe, effective, and usually well-tolerated, so these interventions generally carry a low risk. For TB, this involves prescription of isoniazid (plus pyridoxine) with or without rifampicin (53), and for HBV with entecavir, tenofovir, or lamivudine (54). In individuals with COVID-19 at high epidemiological risk of these infections, our data suggest that there should be a low threshold for screening, and risk-assessment for these prophylactic interventions.

However, it is important to consider the real world practicalities of screening in different settings, and to avoid mandates for screening that cause unintended delays in therapy. This is particularly pertinent for COVID-19 patients who are critically unwell, for whom interventions need to be expedited. For HBV, serum markers (HBsAg, anti-HBc) are generally accessible and affordable, with short turn-around times. For TB screening, interferon gamma release assays (e.g., Quantiferon) and purified protein derivative (PPD) skin tests (e.g., Mantoux) may not be immediately available, and can take 2–3 days to result. Individual risk assessment therefore has to account for the probability of carriage or latent infection, balanced against the potential risks of giving early prophylaxis at the time of TCZ therapy. On these grounds, a decision can be made either to initiate prophylaxis pending results of screening tests (and discontinue prophylaxis if the screen returns negative), or to postpone prophylaxis and initiate only if screening tests return positive.

The high prevalence of HBV exposure and latent TB in some settings pose particular challenges in making balanced risk appraisals. These populations are not adequately represented in research cohorts, and it is therefore not possible to make specific recommendations based on the current literature. Nevertheless, expanding awareness of the potential risk of complications remains important, such that vigilance can be applied, as recommendations are relevant not just at the point of TCZ prescription but during follow-up, with clinical awareness of potential complications in the weeks and months after treatment. As TCZ may blunt markers of infection such as C-reactive protein (CRP), reactivation events may be more difficult to diagnose in this group (1), although increases in CRP are still recognised in response to bloodstream infections (55). In individuals with potential risk of HBV reactivation, monitoring of liver enzymes and HBV markers (HBsAg and viral load), should be considered.

In the case of HCV, flares are possible on biologic therapy and as successful direct acting antiviral (DAA) treatment can now be prescribed, screening is advocated in anyone with risk factors for infection and/or with unexplained abnormalities in liver function tests, such that therapy can be started (51).

### Influence of Blocking IL-6 *in vivo*

IL-6 has an important impact in priming both the humoral and cell-mediated limbs of the adaptive immune system (Figures 1, 2). IL-6 depletion or knock-out experiments in animal models have demonstrated higher pathogen burden, and increased morbidity and mortality, in a range of viral (27, 28), bacterial (28–31), fungal (32), and parasitic infections (33, 34). Abrogating the IL-6 pathway results in alterations in the production of pro-inflammatory cytokines and chemokines, and can influence vascular permeability, the function of the blood-brain barrier (32), and epithelial barrier integrity in the respiratory (29) and urinary (31) tracts. In these *in vivo* experiments, the host immune response can be rescued by administration of recombinant IL-6 (35).

In IL-6 blockade or knock-out, mycobacterial infections are characterised by reduced interferon-gamma production, typically associated with higher bacterial loads, more severe illness and a higher risk of death in mice (56, 57). IL-6 also influences the mileu of other cytokines, including IL-12, IL-23, and type 1 interferons (IFNs), thus playing a role in a complex interaction of factors responsible for co-ordinating macrophage, neutrophil and T-cell activation, with the potential to mediate both pro-inflammatory and anti-inflammatory effects (58, 59) (Figure 2). These roles may have varied influence, depending on the dose and route of inoculum, in different animal hosts, and in acute vs. chronic infection (60). Overall, the effect of IL-6 blockade on outcomes of mycobacterial infection may be modest, and less than that associated with TNF-blockade (61).

Chronic HBV infection is associated with elevated IL-6 levels, both in stable disease (62) and in acute-on-chronic liver failure (63). IL-6 may have a role in HBV control through a variety of mechanisms, including moderation of HBV transcription (64, 65), an influence on the cell surface receptor (NTCP, sodium taurocholate co-transporting polypeptide) (66), and inhibition of the generation of genome-containing nucleocapsids (67), reviewed by Velazquex-Salinas et al. (2). The importance of IL6 in mediating outcomes of HCV infection is demonstrated by an association between IL6 haplotypes and treatment response (68).

### Caveats

We recognise that our estimates of risk are biassed, with a likelihood of under-estimating true risk based on selection of low-risk study participants (including a high proportion of individuals without latent infection, and in some cases completely excluding all at-risk patients), implementation of careful screening and prophylaxis in clinical studies (which differ from usual standard of care), and insufficient durations of follow-up to detect all reactivation events.

Risk appraisal for use of TCZ in COVID-19 is currently based on experience in treating patients with inflammatory and rheumatological disease, who may be at an increased risk of infective complications on the grounds of immunosuppression related to their primary condition, and in whom immunosuppressive therapy is typically prolonged and may involve multiple agents (including steroids, methotrexate and other biological agents). However, although COVID-19 treatment comprises only one or two doses of TCZ, the context of critical illness, combination with steroid therapy, and two doses given in quick succession, may inflate the risks. On all of these grounds, extrapolation of existing TCZ risk assessment data to a population with COVID-19 infection must be undertaken with caution. As the field of COVID-19 therapeutics is expanded, there is interest in using other immunomodulatory drugs (69), which may also be associated with an increase in infective complications.

We were unable to undertake a formal meta-analysis due to the heterogeneity of data, and the reported mean across studies must be interpreted with caution. Further data are needed to investigate the impact of TCZ in racially diverse COVID-19 cohorts including patients with other underlying pathology, followed-up over longer periods (1, 16). This will enable identification of subgroups in whom treatment may be of most benefit, and of the circumstances in which it may carry the most risk. It is notable that many existing publications about the use of TCZ in COVID-19 do not even discuss the potential for these infective complications.

From existing data, we are unable to determine the highest risk period for SIEs with respect to the timing of TCZ dosing, and it is possible that more TB and HBV reactivation events would be identified by longer periods of follow-up, although this trend is not clear from the studies we have identified. However, it is most likely that the greatest risk in COVID-19 therapy is within a short number of weeks of the TCZ dose. Similarly, there are currently no data to determine whether the risk is inflated in individuals receiving two doses rather than one dose of TCZ. Finally, we elected to exclude data on children, due to a current focus on TCZ use to manage severe COVID-19, which arises almost exclusively in adults. However, this should not preclude the collection of robust safety data for TCZ use in children for rheumatological disease.

### Future Questions

There is a need for wider data and more robust guidelines to support the safe use of biologic therapy with appropriate risk assessment and consistent use of antimicrobial prophylaxis, equitable inclusion of diverse populations in research and clinical studies, and ongoing prospective surveillance of patients receiving TCZ to identify complications. In the longer term, better biomarkers that will improve risk stratification, improving the identification of patients at the highest risk of HBV reactivation who stand to gain most from prophylaxis.

The limitations in the data highlight a pressing need for the collection and sharing of complete metadata for clinical studies, including (but not limited to) details of control or comparator groups, use of screening and prophylaxis, and specific dosing regimens. This study also underlines the under-representation of population groups within research studies (by geography or by ethnicity), resulting in substantial uncertainty, which in this case pertains to the very groups likely to be at the highest risk of complications (70).

In addition to the pathogens we have assessed here, there are other SIEs associated with TCZ therapy that remain to be addressed in individuals with COVID-19, including invasive fungal infection, atypical mycobacterial infection, herpes viruses (among which VZV reactivation may be a specific concern), and other opportunistic infections. More data are needed to inform screening, risk assessment, prophylaxis and treatment of these infections in COVID-19 patients. Given that these events are typically uncommon, there is a crucial need for experience to be collated and centralised. The WHO “Vigibase” platform provides a potential foundation for such activity, but collection of enhanced metadata will be required to determine the individuals at risk of SIEs.

## Conclusions

Clinicians prescribing TCZ for patients with COVID should be aware of infective complications, have a low threshold for screening for HBV and TB, recognise the potential role for prophylaxis in those with identified risk factors, and consider the need for surveillance over the weeks and months following therapy. Enhanced awareness of infective complications is important to ensure scrutiny is maintained in patients at the highest risk of adverse outcomes. There is an urgent need for more data in diverse populations in order to inform improved approaches to evidence-based risk assessment and prevention of these potentially serious complications.

## Author Contributions

CC, MIA, PK, and PM conceived the study. CC performed the systematic literature review, with additional review by PM. CC and MAA undertook statistical analysis. OM provided specialist pharmacy input. SM provided expertise in biologic therapy. CC, MAA, and PM generated figures. CC and PM wrote the manuscript. All authors provided editorial input.

## Conflict of Interest

CC receives PhD fellowship funding from GSK. The funder was not involved in the study design, collection, analysis, interpretation of data, the writing of this article or the decision to submit it for publication. The remaining authors declare that the research was conducted in the absence of any commercial or financial relationships that could be construed as a potential conflict of interest.

## Publisher's Note

All claims expressed in this article are solely those of the authors and do not necessarily represent those of their affiliated organizations, or those of the publisher, the editors and the reviewers. Any product that may be evaluated in this article, or claim that may be made by its manufacturer, is not guaranteed or endorsed by the publisher.
