# Supplementary material for: Risk of Reactivation of Hepatitis B Virus (HBV) and Tuberculosis (TB) and Complications of Hepatitis C Virus (HCV) Following Tocilizumab Therapy: A Systematic Review to Inform Risk Assessment in the COVID-19 Era
Source: Front Med (Lausanne). 2021 Aug 20;8:706482. doi: 10.3389/fmed.2021.706482 (PMC8417527; doi:10.3389/fmed.2021.706482)
Supplement: Supplementary file 1 [file Data_Sheet_1.PDF]

SUPPLEMENTARY MATERIAL:

Risk of reactivation of hepatitis B virus (HBV) and tuberculosis (TB) and complications of hepatitis C virus (HCV) following Tocilizumab therapy: A systematic review to inform risk assessment in the COVID era

Cori Campbell, Monique I Andersson, M Azim Ansari, Olivia Moswela, Siraj A Misbah, Paul Klenerman, Philippa C Matthews

CONTENTS:

TABLE S1: Search terms and studies identified through a search of WHO 'VigiBase' database.

TABLE S2: Search terms used in systematic search of Embase, MEDLINE and Web of Science databases.

TABLE S3: PRISMA checklist

TABLE S4: Summary characteristics of observational studies investigating risk of TB infection in individuals receiving tocilizumab.

TABLE S5: Summary characteristics of observational studies investigating risk of HBV infection in individuals receiving tocilizumab.

TABLE S6: Summary characteristics of studies investigating risk of HCV infection in individuals receiving tocilizumab.

TABLE S7: Summary of review articles presenting data on the risks of HBV, HCV and/or TB reactivation in patients on tocilizumab therapy, identified by systematic literature review

Table S1: Results from a search of 'Vigibase', WHO database of Individual Case Safety Reports (ICSRs) / adverse drug reactions (ADRs) ('VigiBase') (https://www.who-umc.org/vigibase/vigibase/) for reports of TB or HBV complications in the context of tocilizumab (TCZ) therapy. Search conducted on 21-Feb-2021. The database reports a total of 47205 records for TCZ, among which 14147 (30%) pertain to 'infections and infestations'.

**Pathogen**	**Complication reported**	**Number reported**
Tuberculosis	Tuberculosis	85
	Pulmonary tuberculosis	29
	Latent tuberculosis[a]	5
	Peritoneal tuberculosis	4
	Disseminated tuberculosis	3
	Lymph node tuberculosis	3
	Pericarditis tuberculosis	2
	Bone tuberculosis	1
	Joint tuberculosis	1
	Extra-pulmonary tuberculosis	1
	Tuberculosis pleurisy	1
	**Total TB complications (% of all reported infection complications)**	**135 (0.9%)**
Hepatitis B virus	Hepatitis B reactivation	25
	Hepatitis B	12
	Viral hepatitis[b]	4
	Acute hepatitis B	1
	**Total HBV complications (% of all reported infection complications)**	**42 (0.3%)**

[a] Latent tuberculosis listed as a complication, but uncertain whether this reflects an episode of reactivation resulting in clinical disease.
[b] Viral hepatitis listed without an aetiological agent; uncertain whether this category includes HBV or other causes of viral hepatitis.

Table S2: Search terms used in systematic search of Embase, MEDLINE and Web of Science databases

Criteria	Database	Search terms
Population	MEDLINE and Embase (searched via Ovid)	(HBV or HCV or "hepatitis B virus" or "hepatitis infection" or "hepatitis C virus" or tuberculosis or TB or "Mycobacterium tuberculosis").af.
	Web of Science	TS=(HBV or HCV or "hepatitis B virus" or "hepatitis infection" or "hepatitis C virus" or tuberculosis or TB or "Mycobacterium tuberculosis")
Exposure	MEDLINE and Embase (searched via Ovid)	(tocilizumab or atlizumab or "interleukin-6 receptor").af.
	Web of Science	TS=(tocilizumab or atlizumab or "interleukin-6 receptor*")
Outcome	MEDLINE and Embase (searched via Ovid)	(infection or reactiv* or exacerbat* or worse*)).ab.
	Web of Science	TS=(infection or reactiv* or exacerbat* or worse*)
Final search	MEDLINE and Embase (searched via Ovid)	((HBV or HCV or "hepatitis B virus" or "hepatitis infection" or "hepatitis C virus" or tuberculosis or TB or "Mycobacterium tuberculosis").af.) AND ((tocilizumab or atlizumab or "interleukin-6 receptor").af.) AND ((infection or reactiv* or exacerbat* or worse*)).af.)
	Web of Science	(TS=(HBV or HCV or "hepatitis B virus" or "hepatitis infection" or "hepatitis C virus" or tuberculosis or TB or "Mycobacterium tuberculosis")) AND (TS=(tocilizumab or atlizumab or "interleukin-6 receptor*")) AND (TS=(infection or reactiv* or exacerbat* or worse*))

MH, medical subject heading term.

Table S3: PRISMA checklist for a systematic review [https://doi.org/10.1136/bmj.b2700]

Section/topic	#	Checklist item	Reported in section
**TITLE**			
Title	1	Identify the report as a systematic review, meta-analysis, or both.	Abstract
**ABSTRACT**			
Structured summary	2	Provide a structured summary including, as applicable: background; objectives; data sources; study eligibility criteria, participants, and interventions; study appraisal and synthesis methods; results; limitations; conclusions and implications of key findings; systematic review registration number.	Unstructured abstract requested by journal.
**INTRODUCTION**			
Rationale	3	Describe the rationale for the review in the context of what is already known.	Included in introduction
Objectives	4	Provide an explicit statement of questions being addressed with reference to participants, interventions, comparisons, outcomes, and study design (PICOS).	Included in introduction
**METHODS**			
Protocol and registration	5	Indicate if a review protocol exists, if and where it can be accessed (e.g., Web address), and, if available, provide registration information including registration number.	No protocol or registration number
Eligibility criteria	6	Specify study characteristics (e.g., PICOS, length of follow-up) and report characteristics (e.g., years considered, language, publication status) used as criteria for eligibility, giving rationale.	Included in methods
Information sources	7	Describe all information sources (e.g., databases with dates of coverage, contact with study authors to identify additional studies) in the search and date last searched.	Included in methods
Search	8	Present full electronic search strategy for at least one database, including any limits used, such that it could be repeated.	Included in suppl table 2
Study selection	9	State the process for selecting studies (i.e., screening, eligibility, included in systematic review, and, if applicable, included in the meta-analysis).	Included in methods
Data collection process	10	Describe method of data extraction from reports (e.g., piloted forms, independently, in duplicate) and any processes for obtaining and confirming data from investigators.	Included in methods
Data items	11	List and define all variables for which data were sought (e.g., PICOS, funding sources) and any assumptions and simplifications made.	Included in methods
Risk of bias in individual studies	12	Describe methods used for assessing risk of bias of individual studies (including specification of whether this was done at the study or outcome level), and how this information is to be used in any data synthesis.	Included in methods (data extraction section)
Summary measures	13	State the principal summary measures (e.g., risk ratio, difference in means).	Incidence of complications is summary measure –

			presented in results and Fig 3
Synthesis of results	14	Describe the methods of handling data and combining results of studies, if done, including measures of consistency (e.g., I^2) for each meta-analysis.	Fig 3

Section/topic	#	Checklist item	Reported on page #
Risk of bias across studies	15	Specify any assessment of risk of bias that may affect the cumulative evidence (e.g., publication bias, selective reporting within studies).	Bias in selection criteria – tables S5 and S6
Additional analyses	16	Describe methods of additional analyses (e.g., sensitivity or subgroup analyses, meta-regression), if done, indicating which were pre-specified.	No additional methods used
**RESULTS**			
Study selection	17	Give numbers of studies screened, assessed for eligibility, and included in the review, with reasons for exclusions at each stage, ideally with a flow diagram.	Presented in results and flow diagram (Fig 2)
Study characteristics	18	For each study, present characteristics for which data were extracted (e.g., study size, PICOS, follow-up period) and provide the citations.	Summarised in tables S5 and S6
Risk of bias within studies	19	Present data on risk of bias of each study and, if available, any outcome level assessment (see item 12).	Final column in tables S5 and S6; specific 'risk of bias' sub-section in results
Results of individual studies	20	For all outcomes considered (benefits or harms), present, for each study: (a) simple summary data for each intervention group (b) effect estimates and confidence intervals, ideally with a forest plot.	Forest plots presented (Fig 3)
Synthesis of results	21	Present results of each meta-analysis done, including confidence intervals and measures of consistency.	Formal meta-analysis not done due to insufficient data, but pooled estimates are presented
Risk of bias across studies	22	Present results of any assessment of risk of bias across studies (see Item 15).	Specific 'risk of bias' sub-section in results, with further comments in discussion section
Additional analysis	23	Give results of additional analyses, if done (e.g., sensitivity or subgroup analyses, meta-regression [see Item 16]).	No additional methods used
**DISCUSSION**			
Summary of evidence	24	Summarize the main findings including the strength of evidence for each main outcome; consider their relevance to key groups (e.g., healthcare providers, users, and policy makers).	Included in discussion

Limitations	25	Discuss limitations at study and outcome level (e.g., risk of bias), and at review-level (e.g., incomplete retrieval of identified research, reporting bias).	Included in discussion
Conclusions	26	Provide a general interpretation of the results in the context of other evidence, and implications for future research.	Included in discussion
**FUNDING**			
Funding	27	Describe sources of funding for the systematic review and other support (e.g., supply of data); role of funders for the systematic review.	Funders included at the end of the manuscript

From: Moher D, Liberati A, Tetzlaff J, Altman DG, The PRISMA Group (2009). Preferred Reporting Items for Systematic Reviews and Meta-Analyses: The PRISMA Statement. PLoS Med 6(7): e1000097. doi:10.1371/journal.pmed1000097. For more information, visit: **www.prisma-statement.org**.

Table S4: Summary characteristics of observational studies investigating risk of TB infection in individuals receiving tocilizumab. Studies reporting incidence of TB reactivation on a per-patient basis are included in the summary figure in the main text (Fig 3A).

Country, Author (Year)	Study design	Population	Follow-up period	Participants receiving tocilizumab, n	TB Cases, n	Age at baseline, years	Sex (% male)	Notes and risk of bias
Hong Kong, Chung (2020) (1)	Retrospective cohort study	Patients with RA or non-specific back pain	11.3 years(med)*	49	1	>60 (med) *	<50 *	Previous episodes of TB infection and the sites of TB infection were recorded, but patients not excluded/included based on this.
Hong Kong, Tang (2020) (2)	Retrospective cohort study	Rheumatic disease patients receiving DMARDs	>=6 months	40	2	53 (mean) *	35*	Participants all underwent either single (TST or IGRA) or dual (TST and IGRA) test for LTBI before starting biologic treatment
Argentina, Cerda (2019) (3)	Retrospective cohort study	Patients with RA, JIA and SpA receiving treatment with anti-TNF, TCZ and/or abatacept	14.8 months (med)*	13	1	52 (mean)*	21*	Only patients with a previous negative TST test were eligible, and all received a second TST test within 22 months of the first test. Patients with history of active TB or LTBI, or those with two tests performed >22 months apart, were excluded.
Taiwan, Lin (2019) (4)	Prospective cohort study	RA patients receiving TCZ	3 years	114	0	59 (med)	15	Patients screened for LTBI and prophylactic isoniazid administered in 12 patients
Hong Kong, Mok (2019) (5) ‡	Retrospective cohort study	RA patients ever treated with DMARDs	3.7 years (mean) *	NA	NA†	54 (mean) *	12*	No information on TB screening
Taiwan, Huang (2018) (6)	Retrospective cohort study	Patients aged >=20 years with immune-mediated inflammatory disorders	19 months (med) *	16	3	NA (80% aged <=65) *	33*	Participants screened for LTBI with IGRA at baseline, those with positive screen offered isoniazid, which a portion of participants refused
India, Malaviya (2018) (7)	Retrospective cohort study	Patients with inflammatory rheumatic musculoskeletal	>= 1 year	7	2	32 (med) *	57*	Patients screened LTBI using augmented Manteau and QFTG test, and positive patients offered prophylaxis

Country, Author (Year)	Study design	Population	Follow-up period	Participants receiving tocilizumab, n	TB Cases, n	Age at baseline, years	Sex (% male)	Notes and risk of bias
		disease receiving biologic DMARDs						
UK, Rutherford (2018) (8)	Prospective cohort study	RA patients receiving biologic therapy	1.8 years (mean)	2171	1	NA	NA	Patients screened according to clinical guidelines at time of treatment initiation (cohort established in 2001)
Italy, Cuomo (2017) (9)	Prospective cohort study	RA patients	10 years (total study duration)*	44	7	55 (mean) *	16*	All patients required negative screen for LTBI at baseline (screened before starting therapy with TST and QFTG assay).
Taiwan, Lim (2017) (10)	Retrospective cohort study	RA patients receiving biologic DMARDs	1.8 years (mean)	31	0	54 (mean) *	26	Patients screened for LTBI via QFTG assay
France, Devauchelle-Pensec (2016) (11)	Prospective open-label study	PMR patients aged 50-80 receiving TCZ	24 weeks (total study duration)	20	0	67 (med)	65	No information on TB screening
France, Morel (2016) (12)	Retrospective cohort study	RA patients receiving TCZ	1.8 years (mean)	1491	1	57 (mean)	NA	No information on TB screening
Argentina, Benzaquen (2015) (13)	Case-control	RA patients receiving biologic DMARDs age- and sex-matched to RA patients receiving non-biologic DMARDs	5 years (total study duration)*	107	NA†	43 (mean) *	21*	No information on TB screening

Country, Author (Year)	Study design	Population	Follow-up period	Participants receiving tocilizumab, n	TB Cases, n	Age at baseline, years	Sex (% male)	Notes and risk of bias
Italy, Cuomo (2015) (14)	Prospective cohort study	RA patients	1 year (total study duration) *	28	1	55 (mean) *	10*	Patients screened negative for LTBI at baseline via TST and QFTG assay; re-screened at one year
France, Gottenberg (2015) (15)	Retrospective cohort study	RA patients treated with abatacept, rituximab or TCZ	1 year (mean)*	1503	0	NA	NA	No information on TB screening
Japan, Yamamoto (2015) (16)	Extension of a single-arm, observational post-marketing surveillance study	RA patients treated with TCZ	3 years (total study duration)	5573	8	59 (mean)	18	Patients screened for TB via chest radiograph, tuberculin test, and interview at baseline, but patients not excluded based on result
Hong Kong, Mok (2014) (17)	Retrospective cohort study	Patients with rheumatic disease receiving biologic DMARDs	1.3 years (mean)	159	0	NA	42*	Routine screening for LTBI using TST. Patients were given isoniazid treatment for LTBI before starting therapy.
Germany, Burmester (2013) (18)	Prospective cohort study	RA patients receiving TCZ	1 year (total study duration)	850	2	56 (mean)	25	No information on TB screening
International, Genovese (2013) (19)	Pooled analysis from 5 RCTs and their open-label extension phases	RA patients included in TCZ randomised controlled trials	3 years (mean)*	2644	9 §	NA	NA	Patients screened for TB and those with active TB were excluded, but those with LTBI received standard of care prophylaxis and were eligible for inclusion
South Africa, Hansrajh (2013) (20)	Open-label extension study	RA patients included in TCZ randomised controlled trial	24 weeks (total observation duration)	29	0	56 (med)	17	Patients screened for LTBI and prophylaxis provided
Japan, Koike (2011) (21)	Prospective post-marketing	RA patients treated with TCZ	28 weeks (total	3881	4	NA	NA	Patients screened for TB via chest radiograph, tuberculin test, and interview before initiation of TCZ

Country, Author (Year)	Study design	Population	Follow-up period	Participants receiving tocilizumab, n	TB Cases, n	Age at baseline, years	Sex (% male)	Notes and risk of bias
	surveillance study		observation duration)					

TB, tuberculosis; RA, rheumatoid arthritis; TCZ, tocilizumab; DMARD, disease-modifying anti-rheumatic drug; TST, tuberculin skin test; IGRA, interferon gamma releasing assay; LTBI, latent tuberculosis infection; JIA, juvenile idiopathic arthritis; SpA, spondyloarthritis; TNF, tumour necrosis factor; QFTG, QuantiFERON®-TB Gold; AS, ankylosing spondylitis; PsA, psoriatic arthritis; PMR, polymyalgia rheumatica.

* Value for whole cohort; value for subset receiving TCZ not reported.

† Authors only report rate of TB infection: 0.36 cases/100 patient-years

‡Conference/published abstract

§ 8 cases thought to be *de novo*

Table S5: Summary characteristics of observational studies investigating risk of HBV reactivation in individuals receiving tocilizumab.

**Country, Author (Year)**	**Study design**	**Population**	**Follow-up period**	**Participants, n**	**HBVr Cases, n**	**Age at baseline, years**	**Sex (% male)**	**Notes and risk of bias**
Taiwan, Kuo (2021) (22)	Retrospective cohort study	RA patients receiving TCZ	9 (med)	97	4	64 (med)	23	Patients were only eligible if they had HBsAg and anti-HBc at baseline. 7 patients were HBsAg+ve. Out of 90 HBsAg-ve 64 were anti-HBc+ve. 3 HBsAg-positive patients who did not receive prophylaxis all experienced reactivation. One anti-HBs-ve patient experienced reactivation
Spain, Rodríguez-Tajes (2021) (23)	Prospective cohort study	Patients with COVID-19	1-1.5 months	44	1	67*	72*	HBsAg+ve patients excluded. Antiviral prophylaxis strongly recommended in all antiHBc+ve patients: 38 out of 69 patients in the cohort received prophylaxis
Taiwan, Lin (2019) (24)	Prospective cohort study	RA patients receiving TCZ	3	114	0	59 (med)	15	Patients screened for HBV and prophylactic NA therapy administered in 11 CHB patients (in whom HBsAg and anti-HBc status was not reported)
Japan, Watanabe (2019) (25)	Retrospective cohort study	RA patients treated with biologic DMARDs	15 months*	25	1	68*	21*	Patients who were anti-HBc and HBsAg-ve and HBV DNA-ve before DMARD initiation were eligible
Korea, Ahn (2018) (26)	Retrospective cohort study	RA patients treated with TCZ	NA (>3 months)	39	0	55 (med)	28	All had HBV serology at baseline. All were HBsAg-negative, and 15 patients had resolved HBV infection (HBsAg-negative; anti-HBc positive).
China, Chen (2017) (27)	Prospective cohort study	RA patients treated with TCZ, with moderate to high disease activity and at least one	12-80 weeks	48	3	46*	21*	Seven patients had CHB and 41 had resolved HBV infection. All cases or HBVr were in CHB (HBsAg+ve) patients.

Country, Author (Year)	Study design	Population	Follow-up period	Participants, n	HBVr Cases, n	Age at baseline, years	Sex (% male)	Notes and risk of bias
		feature of poor prognosis						
Hong Kong, Mok (2014) (17)	Retrospective cohort study	Patients with rheumatic disease receiving biologic DMARDs	1.3 years	159	0	NA	42*	No information for HBV screening
Japan, Nakamura (2014) (28)	Retrospective cohort study	RA patients treated with biologic DMARDs	18 months*	18	2	>=60*	17*	Mix of patients who were HBsAg+ve/-ve and anti-HBc+ve/-ve at baseline. No patients received prophylactic antiviral therapy.

RA, rheumatoid arthritis; HBVr, hepatitis B virus reactivation; HBsAg, hepatitis B serum antigen; HBcAb, hepatitis B core antibody; +ve, positive; -ve, negative; TCZ, tociizumab; CHB, chronic hepatitis B; HBc, hepatitis b virus core antigen; DMARD, disease-modifying anti-rheumatic drugs; DNA, deoxyribonucleic acid; TST, tuberculin skin test.

*Value for whole cohort; value for subset receiving TCZ not reported.

Table S6: Summary of papers reporting an influence of tocilizumab on HCV infection

Author, date of publication	Type of study	Conclusions and/or clinical recommendation(s)
Biehl et al., 2021 (29)	Case series	Flare of HCV responsible for liver injury in 1 of 12 cases of liver injury related to tocilizumab
Sebastiani et al., 2019 (30)	Review and expert consensus recommendations	Recommendations that screening for HCV should be performed in all rheumatoid patients before starting immunosuppressive therapy.
Chiu et al., 2020 (31)	Systematic review	Full text not available
Banerjee et al., 2021 (32)	Network theoretic analysis	Theoretical analysis of the impact of immunosuppressive agents on the network size of the JAK-STAT pathway
Karadag et al., 2016 (33)	Clinical guidelines	Recommendations for any individual with risk factors for HCV infection to be screened prior to biologic therapy, but treatment generally does not cause deterioration (exception is rituximab)
Giannitti et al., 2013 (34)	Case report	TCZ + cyclosporin A treatment for rheumatoid in a patient with HCV was tolerated without any flare in liver enzymes
Song et al., 2019 (35)	Mechanistic study	Investigated the association of IL6 (via the signal transducers and activators of transcription 3 (STAT3) pathway) with HCV infection, but no specific assessment of the impact of TCZ

Table S7: Summary of review articles presenting data on the risks of HBV, HCV and/or TB reactivation in patients on TCZ therapy, identified by systematic literature review

FIrst author and title of paper	Approach	Conclusions and clinical recommendation(s)
Alqahtani, et al., 2021, COVID-19 and hepatitis B infection (36)	Narrative summary of interaction between COVID and HBV infection	• Highlights need for caution regarding potential for HBV reactivation following treatment with TCZ and corticosteroids.<br>• Recommends HBV screening/prophylaxis for patients with elevated transaminases and also in high prevalence populations.
Boyman et al., 2014, Adverse reactions to biological agents and their medical management (37)	Narrative summary of adverse effects of biologic therapy used for treatment of chronic inflammatory and autoimmune disorders	• Reports association between TCZ and increased risk of serious infections, including increased risk of reactivation of latent TB; however, synthesis of literature suggests no overall increase in risk of SIE in patients on TCZ<br>• Recommends TB screening, and subsequent prophylaxis based on risk assessment.<br>• Highest risk of severe infections in the first weeks-months of treatment with biologic agents.<br>• Patients with abnormal LFTs should undergo HBV testing before use of TCZ.
Craig et al., 2018, Gastrointestinal and Hepatic Disease in Rheumatoid Arthritis (38)	Narrative summary of GI complications of Rheumatoid and its treatment	• HBV reactivation recognised as a complication of corticosteroids, but not specifically listed in association with TCZ.<br>• Reactivation of TB recognised, with a higher rate of extrapulmonary disease than in patients not receiving biologic therapy.<br>• No specific recommendations about screening or prophylaxis.
Felis-Giemza et al., 2015, Treatment of rheumatic diseases and hepatitis B virus coinfection (39)	Narrative summary of the HBV disease cycle and points at which biologic disease-modifying anti-rheumatic drugs may influence this cycle	• Highlights exclusion of HBV-infected individuals from most TCZ trials<br>• Recommends consideration of antiviral treatment before TCZ administration in patients with HBsAg seropositivity
Ferro et al., 2017, One year in review 2017: novelties in the treatment of rheumatoid arthritis (40).	Summary of studies published in previous calendar year concerning rheumatoid arthritis treatment novelties	• States that in rheumatoid arthritis patients with high risk of infection or latent TB infection, TCZ is a safer treatment choice compared to other anti-rheumatic drugs

Lai et al., 2019, Useful message in choosing optimal biological agents for patients with autoimmune arthritis (41)	Comparison between biologic agents used alone or in combination for inflammatory / autoimmune arthritis	• TCZ assigned lower risk of TB reactivation compared to anti-TNF therapy for Rheumatoid.<br>• Risk of TB and HBV reactivation recognised with TCZ therapy; TCZ classed as 'intermediate' risk compared to other biologic therapy.
Lunel-Fabiani et al., 2014, Systemic diseases and biotherapies: Understanding, evaluating, and preventing the risk of hepatitis B reactivation (42)	Summary of guidelines for the prevention and management of HBV in patients prescribed biological therapy	• Treatment recommended for all active HBV carriers meeting criteria for therapy<br>• Antiviral prophylaxis for HBsAg carriers who do not meet criteria for therapy (6-12 months)<br>• Inadequate data in many cases to inform strategy for HBsAg negative / anti-HBc positive cases, though prophylaxis is recommended by some guidelines<br>• Vaccination of patients with negative HBV markers is universally recommended
Novosad et al., 2014, Beyond Tumor Necrosis Factor Inhibition: The Expanding Pipeline of Biologic Therapies for Inflammatory Diseases and Their Associated Infectious Sequelae (43)	Summary of data regarding infective complications of biologic therapy	• TB screening is recommended before starting any biologic therapy, with prophylactic therapy in those identified with latent TB.<br>• Screening for HBV and HCV infection recommended before any biologic therapy.<br>• For HBV, give antiviral prophylaxis to those who test HBsAg positive, and close monitoring for HBsAg negative / anti-HBc positive patients with liver function and viral loads
Rose-John et al., 2017 The role of IL-6 in host defence against infections: immunobiology and clinical Implications (44)	Review of biology of IL-6 together with pooled analysis of serious infection events in patients treated with different biological agents	• No specific clinical recommendations are stated, but the paper highlights risks of both HBV and TB reactivation supported by data from clinical trials and pooled analyses.
Sebastiani et al., 2019, Italian consensus recommendations for the management of hepatitis C infection in patients with rheumatoid arthritis (30).	Consensus process produced to review available evidence and produce recommendations regarding HCV management in patients with rheumatoid arthritis	• Screening for HCV should be mandatory before initiation of rheumatoid treatment, and antiviral treatment should be considered in all HCV-infected patients.<br>• HCV eradication should be pursued in all HCV-infected patients, with eradication attempted before initiation of treatment.<br>• Lack of evidence regarding TCZ safety in HCV-infected patients highlighted.

Winthrop et al., 2018 ESCMID Study Group for Infections in Compromised Hosts (ESGICH) Consensus Document on the safety of targeted and biological therapies (45)	Consensus statement based on literature review, summarised into recommendations by expert opinion	• Infection risks associated with TCZ are quantified as similar to anti-TNF therapy.<br>• Screening for HBV should be undertaken prior to starting therapy<br>• Antiviral prophylaxis should be prescribed for HBsAg-positive patients.<br>• Screening should be undertaken for latent TB infection, followed by appropriate prophylaxis or treatment.

anti-HBc - antibody to hepatitis B virus core antigen; ESCMID - European Society of Clinical Microbiology and Infectious Diseases; HBV - hepatitis B virus; HBsAg - hepatitis B surface antigen; HCV - hepatitis C virus; SIE - severe infection events; TB - tuberculosis; TCZ - tocilizumab; TNF - tumour necrosis factor

References:

1. Chung TT, Ko HJ, Lau CS, Chung HY. A retrospective study on the risk of tuberculosis in patients with rheumatoid arthritis. Rheumatol Int [Internet]. 2020;40(6):983–90. Available from: https://doi.org/10.1007/s00296-020-04583-8
2. Tang I, So H, Luk L, Wong V, Pang S, Lao V, et al. Comparison of single and dual latent tuberculosis screening strategies before biologic and targeted therapy in patients with rheumatic diseases: A retrospective cohort study. Hong Kong Med J. 2020;26(2):111–9.
3. Cerda OL, Correa M de los A, Granel A, Marcos AI, Giraldo C, Rillo O, et al. Tuberculin test conversion in patients with chronic inflammatory arthritis receiving biological therapy. Eur J Rheumatol. 2019;6(1):19–22.
4. Lin CT, Huang WN, Hsieh CW, Chen YM, Chen DY, Hsieh TY, et al. Safety and effectiveness of tocilizumab in treating patients with rheumatoid arthritis – A three-year study in Taiwan. J Microbiol Immunol Infect [Internet]. 2019;52(1):141–50. Available from: https://doi.org/10.1016/j.jmii.2017.04.002
5. Mok CC, Wan R, Fong B. AB0425 Prevalence and risk factors of serious infections in rheumatoid arthritis patients receiving the biologic/targeted synthetic dmards: a propensity score analysis from the hong kong biologics registry. In: Annals of the Rheumatic Diseases [Internet]. BMJ; 2019 [cited 2021 Mar 12]. p. 1376.1-1376. Available from: https://ard.bmj.com/content/77/Suppl_2/1376.1
6. Huang SF, Chen MH, Wang F Der, Tsai CY, Fung CP, Su WJ. Efficacy of isoniazid salvage therapy for latent tuberculosis infection in patients with immune-mediated inflammatory disorders – A retrospective cohort study in Taiwan. J Microbiol Immunol Infect [Internet]. 2018;51(6):784–93. Available from: https://doi.org/10.1016/j.jmii.2017.04.001
7. Malaviya AN, Thakaran R, Rawat R, Kapoor S, Garg S, Baghel SS, et al. Real life experience of a screening strategy for latent tuberculosis before treatment with biologicals in indian patients with rheumatic diseases. Indian J Rheumatol. 2018;13(4):233–9.
8. Rutherford AI, Patarata E, Subesinghe S, Hyrich KL, Galloway JB. Opportunistic infections in rheumatoid arthritis patients exposed to biologic therapy: Results from the British Society for Rheumatology Biologics Register for Rheumatoid Arthritis. Rheumatol (United Kingdom). 2018;57(6):997–1001.
9. Cuomo G, D’Abrsca V, Iacono D, Pantano I. The conversion rate of tuberculosis screening tests during biological therapies in patients with rheumatoid arthritis. Clin Rheumatol. 2017;36(2):457–61.
10. Lim CH, Chen HH, Chen YH, Chen DY, Huang WN, Tsai JJ, et al. The risk of tuberculosis disease in rheumatoid arthritis patients on biologics and targeted therapy: A 15-year real world experience in Taiwan. PLoS One. 2017;12(6):1–13.
11. Devauchelle-Pensec V, Berthelot JM, Cornec D, Renaudineau Y, Marhadour T, Jousse-Joulin S, et al. Efficacy of first-line tocilizumab therapy in early polymyalgia rheumatica: A prospective longitudinal study. Ann Rheum Dis. 2016;75(8):1506–10.
12. Morel J, Constantin A, Baron G, Dernis E, Flipo RM, Rist S, et al. Risk factors of serious infections in patients with rheumatoid arthritis treated with tocilizumab in the French Registry REGATE. Rheumatol (United Kingdom). 2017;56(10):1746–54.

13. Benzaquén N, Salinas MH, Pirola JP, Encinas L, Alvarellos AJ, Celina M, et al. Tuberculosis in Patients Treated with Biological Drugs. 2021;2–5.
14. Cuomo G, D’Abrosca V, Russo B, Valentini G. SAT0057 THE CONVERSION RATE OF TUBERCOLOSIS SCREENING TESTS DURING BIOLOGICAL THERAPIES IN PATIENTS WITH RHEUMATOID ARTHRITIS. Ann Rheum Dis. 2015;74(Suppl 2):668.1-668.
15. Gottenberg J-E, Morel J, Ravaud P, Bardin T, Cacoub P, Cantagrel A, et al. OP0035 Tolerance of Rituximab, Abatacept and Tocilizumab in Common Practice: Analysis of the 3 Registries of the French Society of Rheumatology (Air, Ora and Regate). Ann Rheum Dis [Internet]. 2015 Jun 1 [cited 2021 Mar 12];74(Suppl 2):78.2-79. Available from: https://ard.bmj.com/content/74/Suppl_2/78.2
16. Yamamoto K, Goto H, Hirao K, Nakajima A, Origasa H, Tanaka K, et al. Longterm safety of tocilizumab: Results from 3 years of followup postmarketing surveillance of 5573 patients with rheumatoid arthritis in Japan. J Rheumatol. 2015;42(8):1368–75.
17. Mok CC, Chan KY, Lee KL, Tam LS, Lee KW, Tat Chan EY, et al. Factors associated with withdrawal of the anti-TNFα biologics in the treatment of rheumatic diseases: Data from the Hong Kong Biologics Registry. Int J Rheum Dis. 2014;17(s3):1–8.
18. Burmester G-R, Hinüber U von, Richter C, Schwenke H, Gürtler I, Kästner P, et al. FRI0233 TOCILIZUMAB ADMINISTERED IN DAILY CLINICAL PRACTICE - FINAL RESULTS OF THE GERMAN NONINTERVENTIONAL STUDY “ROUTINE.” 2013;(June):2013.
19. Genovese MC, Rubbert-Roth A, Smolen JS, Kremer J, Khraishi M, Gomez-Reino J, et al. Longterm safety and efficacy of tocilizumab in patients with rheumatoid arthritis: A cumulative analysis of up to 4.6 years of exposure. J Rheumatol. 2013;40(6):768–80.
20. Hansrajh V, Rudolph R, Tikly M, Musenge E. P22 An open-label extension study of tocilizumab, in combination with disease modifying anti-rheumatic drugs, in patients with Rheumatoid Arthritis. 2013;32(Suppl 2):S115–S152. Available from: https://cbl.org.lr/doc/cblannualreport2005.pdf
21. Koike T, Harigai M, Inokuma S, Ishiguro N, Ryu J, Takeuchi T, et al. Postmarketing surveillance of tocilizumab for rheumatoid arthritis in Japan: Interim analysis of 3881 patients. Ann Rheum Dis. 2011;70(12):2148–51.
22. Kuo MH, Tseng CW, Lu MC, Tung CH, Tseng KC, Huang KY, et al. Risk of Hepatitis B Virus Reactivation in Rheumatoid Arthritis Patients Undergoing Tocilizumab-Containing Treatment. Dig Dis Sci [Internet]. 2021;(0123456789). Available from: https://doi.org/10.1007/s10620-020-06725-1
23. Rodríguez-Tajes S, Miralpeix A, Costa J, López-Suñé E, Laguno M, Pocurull A, et al. Low risk of hepatitis B reactivation in patients with severe COVID-19 who receive immunosuppressive therapy. J Viral Hepat. 2021;28(1):89–94.
24. Yang HI, Yeh ML, Wong GL et al. Real-World Effectiveness From the Asia Pacific Rim Liver Consortium for HBV Risk Score for the Prediction of Hepatocellular Carcinoma in Chronic Hepatitis B Patients Treated With Oral Antiviral Therapy - PubMed. J Infect Dis [Internet]. 2020 [cited 2020 Jul 1];221(3):389–99. Available from: https://pubmed.ncbi.nlm.nih.gov/31550363/

25. Watanabe T, Fukae J, Fukaya S, Sawamukai N, Isobe M, Matsuhashi M, et al. Incidence and risk factors for reactivation from resolved hepatitis B virus in rheumatoid arthritis patients treated with biological disease-modifying antirheumatic drugs. Int J Rheum Dis. 2019;22(4):574–82.
26. Ahn SS, Jung SM, Song JJ, Park YB, Park JY, Lee SW. Safety of tocilizumab in rheumatoid arthritis patients with resolved hepatitis B virus infection: Data from real-world experience. Yonsei Med J. 2018;59(3):452–6.
27. Chen MH, Chen MH, Liu CY, Tsai CY, Huang DF, Lin HY, et al. Hepatitis B virus reactivation in rheumatoid arthritis patients undergoing biologics treatment. J Infect Dis. 2017;215(4):566–73.
28. Nakamura J, Nagashima T, Nagatani K, Yoshio T, Iwamoto M, Minota S. Reactivation of hepatitis B virus in rheumatoid arthritis patients treated with biological disease-modifying antirheumatic drugs. Int J Rheum Dis. 2016;19(5):470–5.
29. Biehl A, Harinstein L, Brinker A, Glaser R, Muñoz M, Avigan M. A case series analysis of serious exacerbations of viral hepatitis and non-viral hepatic injuries in tocilizumab-treated patients. Liver Int. 2020;(December 2020):515–28.
30. Sebastiani M, Milazzo L, Atzeni F, Vacchi C, Manfredi A, Quartuccio L, et al. Italian consensus recommendations for the management of hepatitis C infection in patients with rheumatoid arthritis. Mod Rheumatol [Internet]. 2019;29(6):895–902. Available from: https://doi.org/10.1080/14397595.2018.1558918
31. Chiu YM, Chen DY. Infection risk in patients undergoing treatment for inflammatory arthritis: non-biologics versus biologics. Expert Rev Clin Immunol. 2020;16(2):207–28.
32. Banerjee A, Goswami RP, Chatterjee M. Network theoretic analysis of JAK/STAT pathway and extrapolation to drugs and viruses including COVID-19. Sci Rep [Internet]. 2021;11(1):1–6. Available from: https://doi.org/10.1038/s41598-021-82139-x
33. Karadag O, Kasifoglu T, Ozer B, Kaymakoglu S, Kus Y, Inanc M, et al. Viral hepatitis screening guideline before biological drug use in rheumatic patients. Eur J Rheumatol. 2016;3(1):25–8.
34. Giannitti C. Efficacy and safety of tocilizumab combined with cyclosporine A in a patient with rheumatoid arthritis and concomitant chronic hepatitis C virus infection. Theory Probab its Appl. 2013;56(1):178–9.
35. Song Y, Yang X, Shen Y, Wang Y, Xia X, Zhang AM. STAT3 signaling pathway plays importantly genetic and functional roles in HCV infection. Mol Genet Genomic Med. 2019;7(8):1–19.
36. Alqahtani SA, Buti M. COVID-19 and hepatitis B infection Review COVID-19 and hepatitis B infection. 2021;
37. Boyman O, Comte D, Spertini F. Adverse reactions to biologic agents and their medical management. Nat Rev Rheumatol. 2014;10(10):612–27.
38. Craig E, Cappelli LC. Gastrointestinal and Hepatic Disease in Rheumatoid Arthritis [Internet]. Vol. 44, Rheumatic Disease Clinics of North America. W.B. Saunders; 2018 [cited 2021 Mar 16]. p. 89–111. Available from: /pmc/articles/PMC5726432/
39. Felis-Giemza A, Olesińska M, Świerkocka K, Więsik-Szewczyk E, Haładyj E. Treatment of rheumatic diseases and hepatitis B virus coinfection. Rheumatol Int. 2015;35(3):385–92.
40. Ferro F, Elefante E, Luciano N, Talarico R, Todoerti M. One year in review 2017: Novelties in the treatment of rheumatoid arthritis. Clin Exp Rheumatol. 2017;35:03721–0734.

41. Lai JH, Ling XC, Ho LJ. Useful message in choosing optimal biological agents for patients with autoimmune arthritis. Biochem Pharmacol [Internet]. 2019;165(January):99–111. Available from: https://doi.org/10.1016/j.bcp.2019.03.007
42. Lunel-Fabiani F, Masson C, Ducancelle A. Systemic diseases and biotherapies: Understanding, evaluating, and preventing the risk of hepatitis B reactivation. Jt Bone Spine. 2014;81(6):478–84.
43. Novosad SA, Winthrop KL. Beyond tumor necrosis factor inhibition: The expanding pipeline of biologic therapies for inflammatory diseases and their associated infectious sequelae. Clin Infect Dis. 2014;58(11):1587–98.
44. Rose-John S, Winthrop K, Calabrese L. The role of IL-6 in host defence against infections: Immunobiology and clinical implications. Nat Rev Rheumatol. 2017;13(7):399–409.
45. Winthrop KL, Mariette X, Silva JT, Benamu E, Calabrese LH, Dumusc A, et al. ESCMID Study Group for Infections in Compromised Hosts (ESGICH) Consensus Document on the safety of targeted and biological therapies: an infectious diseases perspective (Soluble immune effector molecules [II]: agents targeting interleukins, immunoglobuli. Clin Microbiol Infect. 2018;24:S21–40.
